# Ultra-Processed Foods Are the Major Sources of Total Fat, Saturated and Trans-Fatty Acids among Tunisian Preschool and School Children: A Cross-Sectional Study

**DOI:** 10.3390/children9020126

**Published:** 2022-01-19

**Authors:** Darine Dogui, Radhouene Doggui, Ayoub Al-Jawaldeh, Jalila El Ati, Myriam El Ati-Hellal

**Affiliations:** 1 Nutrition Surveillance and Epidemiology in Tunisia Research Laboratory, National Institute of Nutrition and Food Technology, Tunis 1007, Tunisia; deryn_dogui@yahoo.fr (D.D.); doggui.radhouene@gmail.com (R.D.); jalila.elati@yahoo.fr (J.E.A.); 2 Centre de Formation Médicale du Nouveau-Brunswick, Université de Sherbrooke, Moncton, NB E1A 7R1, Canada; 3 Department of Family and Emergency Medicine, Université de Sherbrooke, Sherbrooke, QC J1K 2R1, Canada; 4 Regional Office for the Eastern Mediterranean (EMRO), World Health Organization (WHO), Cairo 7608, Egypt; aljawaldeha@who.int; 5 Laboratory Materials Molecules and Applications, Preparatory Institute for Scientific and Technical Studies, University of Carthage, Tunis 2070, Tunisia

**Keywords:** trans fatty acids, saturated fatty acids, ultra-processed foods, children, Tunisia

## Abstract

Excessive fat and fatty acids intake are associated with significant health hazards such as obesity or chronic diseases. This study aimed to provide the first data on total fat, saturated fatty acids (SFA) and trans fatty acids (TFA) intakes and their major food sources in Tunisian children. A total of 1200 children, aged 3 to 9 years old, were randomly selected from primary schools and kindergartens under a cross-sectional design. The 24-h dietary recall method and diet history for the month preceding the survey were used to assess dietary intake. The energy percentages of total fat, SFA and TFA in Tunisian children were 29.6%, 11.4% and 0.15%, respectively. No sex differences were found. The WHO recommendations for total fat, SFA and TFA were adopted by 58%, 39% and 89% of the study population, respectively. The leading food groups of fat and fatty acids were ultra-processed foods, breakfast cereals and dairy products. The meat, fish, eggs, and fish alternatives were the fifth main contributors to Tunisian children’s total fat and SFA intakes. The implementation of a relevant strategy for fat reduction, especially from ultra-processed foods, considered as low nutrient energy-dense products, is needed to promote health among children and prevent diet-related chronic diseases.

## 1. Introduction

Fatty acids are carboxylic acids with either saturated or unsaturated aliphatic chains [1,2,3]. Saturated fatty acids (SFA) have no double bonds, while unsaturated fatty acids have at least one double bond in their cis or trans configuration [4]. The main sources of SFA in the food supply are animal products, including meat and dairy products and processed foods, e.g., biscuits and cakes [2,5,6]. Trans fatty acids (TFA) are produced naturally in ruminants’ stomachs or industrially by partial hydrogenation of vegetable oils. Hydrogenation increases the melting point of fats, making it possible to convert fats from the liquid state to the semi-solid or solid-state [7,8]. The benefits of such a process are the increase of flavor stability and shelf life of unsaturated fatty acids.

The association between high dietary intakes of fat and mortality remains controversial. In this way, the main findings of a meta-analysis carried out on six randomized controlled trials which examined the association between dietary fat, serum cholesterol, and the risk of coronary heart disease (CHD) indicated there was no statistically significant difference in deaths from CHD between the intervention and control groups [9]. By contrast, several studies showed that the consumption of diets high in SFA increased the risk of mortality from all causes, cardiovascular disease (CVD), and cancer, and high dietary intakes of TFA were associated with higher all-cause mortality and CVD [8,10,11]. Because of these deleterious health effects, 2018 WHO draft guidelines on SFA and TFA intake for adults and children recommend reducing the intake of SFA and TFA to less than 10% and 1% of total energy intake, respectively. They suggest using polyunsaturated fatty acids as a replacement energy source, if needed [12].

Given the long-term effect of childhood dietary consumption on adult health and the risks associated with sustained high intake of SFA and TFA, this study aimed to describe eating patterns and find the leading food group sources of these fatty acids in Tunisian preschool school-age children.

## 2. Material and Methods

### 2.1. Subjects and Study Design

The subjects of this study were a sample of 1200 children aged 3–9 years old, randomly selected from primary schools and kindergartens in the Greater Tunis region from April to May 2017. This region is mainly an urban area (about 2.8 million inhabitants, of whom 93% live in urban areas and 7% in rural areas) and includes four governorates (Tunis, Manouba, Ariana and Ben Arous). A two-stage clustered sampling was designed by the National Institute of Statistics. Stratification was made depending on each of the four governorate and urban/rural environments. At the first level, 30 primary schools and 30 kindergartens were selected from the initial sampling frame. At the second level, 20 children were systematically drawn from each educational institution.

### 2.2. Dietary Intake Assessment

Children’s dietary intake was assessed face-to-face with mothers or caretakers for children during home visits using 24-h dietary recall and the diet history for the month preceding the survey [13]. Trained dieticians recorded data on the types of all foods and drinks consumed by children. A detailed and precise description of food items was made using household tableware, photos and known weight of food portions [14]. The energy and nutritional content of consumed food items and recipes were estimated by laboratory analysis, the Tunisian food composition table [11], the USDA table [15] and the food processor software [16]. The adequacy of nutrient intake was assessed using the dietary reference intakes of the WHO [15].

### 2.3. Laboratory Nutrient Analysis

The most commonly processed foods consumed by children were purchased from retail outlets and analyzed in the laboratory using gas chromatography-mass spectrometry (GC-MS) with hydrolytic extraction. The revised version of the Association of Official Agricultural Chemists official method 996.06 was adopted for total fat, SFA and TFA analysis [17].

### 2.4. Anthropometric Assessment

Anthropometric measurements of children followed standard procedures [18]. Standing height was measured to the nearest 0.1 cm with the use of a wall-mounted stadiometer (Person-check^®^, Kirchner and Wilhelm, Germany), weight was measured to the nearest 0.1 kg on a calibrated scale (Detecto, Webb City, MO, USA). Body Mass Index = weight/height^2^ for-age z-scores were derived from the WHO reference for children [19] and used to define underweight (BMI-for-age Z-score < −2), overweight (BMI-for-age Z-score > +1) and obesity (BMI-for-age Z-score > +2). Stunting was defined as a height-for-age Z-score <−2) [19].

### 2.5. Socioeconomic and Demographic Characteristics

Data on the level of education and occupation of children’s mother and the head of household were collected by questionnaire. The economic level of the household was assessed using an asset-based proxy and classified as low, medium or high according to tertiles of this index [13,20].

### 2.6. Ethics Approval and Consent to Participate

All applicable institutional and governmental regulations concerning the ethical use of human volunteers were respected during this study. The survey protocol was reviewed and approved by the Tunisian National Council of Statistics (Visa n°3/2017) and the Ethical Consultative Committee of the National Institute of Nutrition and Food Technology. After being thoroughly informed of the survey’s purpose, requirements, and procedures, all parents gave their verbal consent and children gave their assent (those aged six years and above). All data were de-identified during analysis.

### 2.7. Data Management and Statistical Analysis

Data entry was carried out in duplicate using Epidata software version 3.1 [21]. Data analysis was performed by Stata 14 software [22], taking into account the complex sampling design (including stratification, clustering, sampling weights and post-stratification on sex, age and place of residence) and using svy Stata commands specific to survey data analysis [23]. The normality of the distribution was tested using the Shapiro-Wilk test. Descriptive results are expressed as means for interval variables, and as proportions for categorical variables. Linear regression was used to examine the association of categorical variables (sex and age classes) with nutrient concentration, while proportion comparison was done by the Chi—squared test. The significance level was set to 0.05.

## 3. Results

Among the 1200 eligible children, 20 refused, 14 were absent and five were ill. Thus, the response rate was 97% and the sample under study had no missing value. The characteristics of the study population according to gender are presented in Table 1.

The sample was evenly distributed among household economic levels. Approximately all household heads have a profession, while half of the mothers do not work. Over two-thirds of household heads and mothers have a high school or university education. A proportion of 60% of the children were of normal body weight, with about 26% overweight and 10% obese.

The mean daily total fat, SFA and TFA intakes of boys and girls of all age groups are reported in Table 2. The percentage total fat energy of Tunisian children aged 3 to 9 years old was 29.6%. The mean SFA and TFA intakes of the studied population were 11.4 (% E) and 0.15 (% E), respectively. No sex differences were found. According to age, children aged 3 to 4 years old had significantly higher SFA (11.7% E) and TFA (0.18% E) intakes than the other age groups (*p* < 0.0001).

In addition, Table 3 presents the percentage of children meeting the WHO recommendations for total fat, SFA and TFA according to gender. Up to 58% of the study population adhered to the WHO recommendations for total fat intake. In 41% of the children, total fat intake was higher than 30% E. Only 39% of the children were in compliance with the SFA recommendations. A high proportion of the children aged 3–9 years old (89%) had an adequate TFA intake (<1% E). No gender differences were observed.

The percentage contributions of the major food groups to the fat and fatty acids intake in the total study population can be found in Table 4. Ultra-processed foods (mainly cheese, package cakes, pies and biscuits) were the major food sources of total fat, SFA and TFA intakes in Tunisian children with respective percentage contributions of 32.5%, 28.9% and 48.4%. Breakfast cereals were the second and the third main contributors to the total fat and SFA consumption, respectively. Dairy products were classified at the second and the fourth rank respectively for fatty acids and total fat intakes. Beverages and industrial juices did not contribute to the fat and fatty acids intake.

## 4. Discussion

In the present study, we reported for the first time the intake of total, saturated and trans-fatty acids and their major food sources among 3–9 years old Tunisian children using a cross-sectional survey. We found that the mean intake of total fat falls within the WHO recommendations, but a large proportion of the population (41%) exceeded the recommended limit of 30% E. SFA intake in almost two-thirds of the children was greater than 10% E. However, the TFA consumption was under the WHO recommendations for nearly all of them [15]. Compared to findings on total fat and SFA intake of children and adolescents in other countries, our results are higher than those reported in Korea [24], Mexico [25] or Japan [26], similar to those found in Guatemala [27] or US [28] and lower than results registered in European countries where the mean total fat intake was 33.3% E, with a mean SFA intake of 13.8% E [29]. The consumption of TFA by Tunisian children was very low compared to data registered elsewhere. Monge-Rojas et al. (2013) reported a mean TFA intake of 1.3% E in Costa Rican adolescents [30], while the average dietary intake of TFA in Spanish children aged 4–5 years old was 1.36 g/d which corresponds to 0.77% E [31]. Results from Canadian children aged 5–6 years old showed a mean TFA intake of 0.71% E [32]. These results are expected because the overall levels of TFA in most processed food products available on the Tunisian market are low (<1 g/100 g of sample), except in margarine (5.56 g/100 g).

Our results revealed that ultra-processed foods (mainly cheese and cakes, pies and biscuits) were the greatest source of fat and fatty acids in Tunisian children, followed by breakfast cereals for total fat and dairy products for fatty acids. Ultra-processed foods are food products formulated mainly or entirely from processed ingredients, including little or no whole foods [33]. The early consumption of these products could lead to adverse health effects such as obesity or chronic diseases [34,35]. Therefore, it is important to understand the role of food processing and to formulate public health strategies to reduce the consumption of ultra-processed products early in life. Comparing food sources of fat and fatty acids is not easy because food groupings differ between the research studies. The definition of the food groups in the present study was based on the Tunisian food composition table and the USDA table [11,15]. The important contributions of ultra-processed foods, breakfast cereals, and dairy products in children and adolescents’ fat and fatty acids intake were also found elsewhere. Asakura and Sasaki (2017) reported that meat, dairy products, and confectionery were the three major sources of SFA in Japanese schoolchildren (26.4%, 25.7% and 11.3% of total SFA intake) [26]. According to Wang et al. (2018), meat, poultry and fish, milk and mixtures consisting mainly of grain were the leading food sources of saturated fats in US children [28]. The Korean study revealed that milk was the major food source of total fat and SFA in 3–5 years old children, with respective percentages contributions of 15.6% and 29.5%, followed by pork and eggs [24]. In Costa Rica, bakery products, red meat and dairy products were the main contributors to SFA and TFA intakes in adolescents [30], while fried eggs, whole milk, breakfast cereals and fresh cheese were among the major food sources of total fat and SFA in diets of Guatemalan schoolchildren [27]. The principal food groups contributing to the total TFA intake in Spanish children were milk (21%), processed baked goods (16%), sweets (12%), fast food (12%) and white bread (10%) [31]. These were comparable to those reported in the Canadian study [32]. Generally, the top three food groups contributing to the total fat and SFA intakes in European adolescents were meat, fish, eggs and meat alternatives (mainly meat), low-nutrient, energy-dense foods (mainly cakes, pies and biscuits) and dairy and soy products (mainly cheese) [29]. In our study, the meat, fish, eggs and fish alternatives were the fifth main contributors to the total fat and SFA intakes in Tunisian children, with respective percentages of 10.7% and 10.8%. This result is probably due to differences in dietary habits between the Tunisian and other world populations. In Tunisia, the average annual meat consumption per capita was around 32.5 kg in 2015, close to the global average of 34.3 kg, but far from 69 kg in the European Union and 98.3 kg in the United States [36,37]. On the other hand, the mean annual consumption of lean meat in 2015 (19.4 kg for poultry and white meat) is much more important than the consumption of fatty meat (7.1 kg for sheep meat and 3.1 kg for bovine meat) [36]. The general food price index, which makes sheep and bovine meat proportionately more expensive than the other food products, could explain this trend of meat consumption among Tunisian people [38].

Given that a large proportion of Tunisian children exceeded the recommended levels of total fat and SFA intake, the implementation of several policy actions is necessary to prevent diseases and promote health in Tunisia. In this context, the WHO regional office has developed policy guidance with recommended actions for countries in the Eastern Mediterranean Region to reduce national fat intake. These recommendations include establishing mandatory labelling schemes for SFA content that are easily understandable for most consumers and replacing industrially-produced TFA with healthier oils and fats [39]. Future health policies should focus primarily on reducing the children’s intake of ultra-processed foods and increasing access to high nutritive quality foods such as vegetables, fruits, whole-grain products and animal source foods with health-promoting fats (e.g., fish) [40].

## 5. Strengths and Limitations of the Study

A strength of our study is the detailed analyses of total fat, SFA and TFA intakes and their major food sources in children in a middle-income country. In addition, the used 24-h dietary recall coupled with dietary history and estimated portions is appropriate to assess usual food intake. However, the cross-sectional study relies on the respondent’s memory, which can lead to recall bias.

## 6. Conclusions

Since 41% and 61% of Tunisian children consumed excess total fat and SFA, respectively, rapid intervention is needed for fat reduction in the Tunisian population. Intake of TFA was relatively low compared to other research studies. Nevertheless, elimination of industrial TFA is strongly recommended due to its association with increased risk of heart attack and death. The major dietary sources of total fat SFA and TFA were ultra-processed foods, breakfast cereals, and dairy products in Tunisian children. As ultra-processed foods are considered as low nutrient and dense energy foods, public health nutrition efforts should continue to reduce the consumption of these products and promote the intake of healthy diets.

## Figures and Tables

**Table 1:** Characteristics of Tunisian children aged 3–9 years old.

Physiological Characteristics	All		Boys		Girls	
	*n*	% ^a^	*n*	%	*n*	% ^a^
Boys	582	50				
Girls	582	50				
**Age (years)**						
3–4	350	33.8	191	36.5	159	31.0
5–6	334	29.9	162	28.7	172	31.0
7–8	307	23.2	149	22.6	158	23.9
9–10	173	13.1	80	12.2	93	14.1
**Socio-economic factors**						
**Economic level of the household**						
Upper tertile	383	32.2	187	31.5	196	32.9
Medium tertile	392	34.1	197	34.0	195	34.1
Lower tertile	389	33.7	198	34.5	191	33.0
**Profession of household head**						
Upper/medium	507	44.4	247	42.9	260	45.8
Employee/worker	637	54.0	324	55.3	313	52.8
Not working/retired	20	1.6	11	1.8	9	1.4
**Education of household head**						
University/Secondary	882	76.4	440	75.9	442	76.9
Primary school or none	282	23.6	142	24.1	140	23.1
**Profession of mother**						
Upper/medium	332	29.5	160	28.8	172	30.2
Employee/worker	253	22.0	123	21.4	130	22.7
Not working/retired	579	48.5	299	49.8	280	47.1
**Education of mother**						
University/Secondary	878	76.5	433	75.4	445	77.6
Primary school or none	286	23.5	149	24.6	137	22.4
**Anthropometric characteristics**						
Stunting	16	1.4	10	1.7	6	1.0
Underweight	37	3.0	19	3.1	18	2.9
Overweight	311	26.0	151	25.2	160	26.7
Obesity	122	9.9	65	10.7	57	09.1

^a^ Weighted percentage.

**Table 2:** Intake of total fat, SFA and TFA according to gender and age by Tunisian children aged 3–9 years old.

Nutrient	Unit		Total	Gender			Age Groups				
				Boys	Girls	*p* Value	3–4 Years Old	5–6 Years Old	7–8 Years Old	9–10 Years Old	*p* Value ^a^
Fat total	(g/d)	Mean (s.e.) ^b^	49.8 (0.5)	50.5 (0.7)	48.9 (0.6)	0.084	46.3 (0.8)	51.0 (1.0)	52.3 (0.9)	50.9 (1.2)	0.000
		95% CI	48.7–49.7	49.1–51.9	47.6–50.1		44.7–47.8	49.0–52.9	50.6–54.1	48.5–53.3	
	(% E) ^c^	Mean (s.e.)	29.6 (0.3)	29.6 (0.6)	29.6 (0.3)	0.931	29.2 (0.3)	30.4 (1.0)	29.3 (0.4)	29.2 (0.5)	0.968
		95% CI	28.9–30.2	28.5–30.8	29.0–30.1		28.5–29.9	28.5–32.3	28.6–30.1	28.3–30.2	
SFA ^d^	(g/d)	Mean (s.e.)	19.2 (0.2)	19.6 (0.3)	18.8 (0.3)	0.070	18.5 (0.4)	19.7 (0.4)	19.6 (0.4)	19.4 (0.6)	0.110
		95% CI	18.8–19.7	19.0–20.2	18.2–19.4		17.8–19.2	18.9–20.5	18.7–20.4	18.1–20.7	
	(% E)	Mean (s.e.)	11.4 (0.1)	11.3 (0.1)	11.4 (0.2)	0.887	11.7 (0.2)	11.5 (0.2)	10.9 (0.2)	11.0 (0.3)	0.008
		95% CI	11.2–11.6	11.1–11.6	11.1–11.7		11.3–12.0	11.2–11.9	10.5–11.3	10.5–11.5	
TFA ^e^	(g/d)	Mean (s.e.)	0.24 (0.01)	0.26 (0.02)	0.23 (0.02)	0.158	0.29 (0.03)	0.25 (0.03)	0.24 (0.03)	0.13 (0.02)	0.000
		95% CI	0.22–0.27	0.22–0.30	0.19–0.26		0.23–0.34	0.20–0.30	0.18–0.29	0.08–0.18	
	(% E)	Mean (s.e.)	0.15 (0.01)	0.16 (0.01)	0.14 (0.01)	0.219	0.18 (0.02)	0.15 (0.02)	0.14 (0.02)	0.08 (0.01)	0.000
		95% CI	0.13–0.16	0.13–0.18	0.11–0.16		0.15–0.22	0.12–0.18	0.10–0.17	0.05–0.10	

^a^—Comparison between sexes adjusted for age. ^b^—Weighted mean value (standard error). ^c^—Energy percent. ^d^—Saturated fatty acids. ^e^—Trans fatty acids.

**Table 3:** Percentage of Tunisian children adhering to WHO recommendations for fat, SFA and TFA by gender.

Nutrient (% E)	Total (*n* = 1164)	Boys (*n* = 582)	Girls (*n* = 582)	*p* Value
Total fat				
<15	<1	<1	<1	0.759
15–30 ^a^	58	59	57	
>30	41	40	42	
SFA ^b^				
<10 ^a^	39	40	38	0.42
≥10	61	60	62	
TFA ^c^				
<1 ^a^	89	89	89	0.945
≥1	11	11	11	

^a^ Recommended levels of total fat, SFA and TFA according to WHO. ^b^ Saturated fatty acids. ^c^ Trans fatty acids.

**Table 4:** Percentage contributions of the major food groups to the total fat, SFA and TFA intakes in Tunisian children.

Total Fat			SFA ^a^			TFA ^b^		
Rank	Food Group	% ^c^	Rank	Food Group	%	Rank	Food Group	%
1	Ultra-processed foods	32.5	1	Ultra-processed foods	29.0	1	Ultra-processed foods	48.4
2	Breakfast cereals	20.5	2	Dairy products	22.7	2	Dairy products	47.1
3	Vegetables, legumes and fruits	16.1	3	Breakfast cereals	17.3	3	Fat and oils	4.4
4	Dairy products	11.7	4	Vegetables, legumes and fruits	12.9	4	Breakfast cereals	0.1
5	Meat, fish and eggs	10.7	5	Meat, fish and eggs	10.8	5	Beverages and industrial juices	0.0
6	Fat and oils	5.8	6	Fat and oils	5.2	6	Meat, fish and eggs	0.0
7	Potatoes and grains	2.0	7	Potatoes and grains	1.6	7	Potatoes and grains	0.0
8	Beverages and industrial juices	0.2	8	Beverages and industrial juices	0.2	8	Vegetables, legumes and fruits	0.0

^a^ Saturated fatty acids. ^b^ Trans fatty acids. ^c^ Percentage contributions of food groups.

## Data Availability

The data is available from the corresponding author upon a reasonable request.
